# Metagenomic analysis of the microbiome of lung adenocarcinoma with pure ground‐glass opacity

**DOI:** 10.1002/ctm2.698

**Published:** 2022-01-21

**Authors:** Zhenyu Zhao, Banglun Qian, Xiong Peng, Wei Yin, Qidong Cai, Pengfei Zhang, Boxue He, Shuai Shi, Weilin Peng, Guangxu Tu, Yongguang Tao, Xiang Wang, Fenglei Yu, Yunping Li

**Affiliations:** ^1^ Department of Thoracic Surgery The Second Xiangya Hospital of Central South University Changsha China; ^2^ Hunan Key Laboratory of Early Diagnosis and Precise Treatment of Lung Cancer The Second Xiangya Hospital of Central South University Changsha China; ^3^ Key Laboratory of Carcinogenesis and Cancer Invasion, Ministry of Education, Department of Pathology, Xiangya Hospital Central South University Changsha China; ^4^ NHC Key Laboratory of Carcinogenesis (Central South University) Cancer Research Institute and School of Basic Medicine Central South University Changsha China; ^5^ Department of Ophthalmology The Second Xiangya Hospital of Central South University Changsha China

Dear Editor,

In this research, we firstly explored the role of the microbiome combined with transcriptomics and proteomics in pure ground‐glass opacity (pGGO) (<1 cm) development by 16S rRNA gene sequencing technology (Figure S1), and demonstrated the pGGO microbiome features in lung adenocarcinoma (LUAD) patients. Notably, through our study, the microbiome in pGGO was mainly involved in immunosuppression, inflammation and angiogenesis processes, and the Oxalobacteraceae was the specific microbe in the pGGO tissues.

GGO, the hazy opacity that does not obscure underlying bronchial structures or pulmonary vessels, was often proved to be the early stage of the LUAD in our clinical centre. Recent studies had shown that the size of GGO (<1 cm) is a risk factor for the occurrence and development of LUAD.^1^ Therefore, an in‐depth exploration of GGO has great significance for the study of the pathological mechanism of LUAD. The microbiome is often considered the ‘forgotten organ’, and microbial products could affect inflammation and tumour progression, which in turn affect commensal disequilibrium.^2^ In this research, all patients were diagnosed with pGGO (<1 cm) by high‐resolution computed tomography examination and underwent surgical resection with curative intent, meanwhile, pathological analyses and staging were completed according to the revised AJCC 8th tumor‐node‐metastasis guidelines classification (the clinical data are provided in Table S1). There were 8, 30 and 6 specimens (paired pGGO tissues and paracancerous tissues) collected for metagenomic, transcriptomics and proteomics sequencing, respectively. The results of the 16s rDNA sequencing technology identified that there were 1053 operational taxonomic units (OTUs) in pGGO. Following preset selection criteria (*log |FC| > 2*, *p‐*value <.05), we identified five differential bacteria OTUs between pGGO and pGGO paracancerous tissues (Figure 1A, Table S2; up‐regulated: 3; down‐regulated: 2). Next, we explored the function and molecular mechanisms of five specific bacteria OTUs in pGGO combined with transcriptomics and proteomics. Then, we identified five differential expressed proteins (DEPs) that were strongly associated with differential OTUs (Table S3, correlation coefficient* > *.9*, log |FC| *> 1, up‐regulated: 0; down‐regulated: 5). The gene ontology enrichment pathway analysis suggested that five DEPs were significantly enriched in leukocyte activation involved in immune response, transport and angiogenesis pathways, *ABCA13*, *LRG1*, and *TRAPPC3* were the core nodes and these genes were down‐regulated in these biological processes (Figure 1B). Thus, the five differential OTUs may involve leukocyte activation in immune response, transport and angiogenesis biological processes of pGGO by interacting with *ABCA13*, *LRG1* and *TRAPPC3*, and influencing the five DEPs. Similarly, 37 differential expressed genes (DEGs) (up‐regulated: 15; down‐regulated: 22) were strongly associated with differential OTUs when correlation coefficient >.9, log |FC| > 1 (Figure 1C, Table S4). The gene ontology (GO) analyses showed that the DEGs were significantly enriched in immune‐related processes, such as neutrophil‐mediated cytotoxicity (Figure 1D). The functional network suggested that 37 DEGs were significantly involved in Hedgehog signaling pathways, ECM receptor interaction, and so on (Figure 2A). The expression of 10 significant selected DEGs was confirmed by real‐time quantitative polymerase chain reaction (PCR) with 16 paired normal and GGO tissues (Figure 2B–K). Notably, these results suggest that the OTUs inhibit the Hedgehog signalling pathway by repressing Indian hedgehog gene (*IHH*) expression, leading to immunosuppression. Based on the recent study, *IHH* was reported as an inducing factor in cancer that restricts tumour cell proliferation and suppresses activation of the immune system through Hedgehog signalling or Wnt signalling pathways.^3^ Thus, five differential bacteria OTUs may suppress the regulation of the immune system by interacting with *IHH* or other genes, which in turn predisposes the tumour‐promoting inflammation process, and inhibit the tumour cell death or promote the proliferation and differentiation of tumour cells.

**FIGURE 1:**
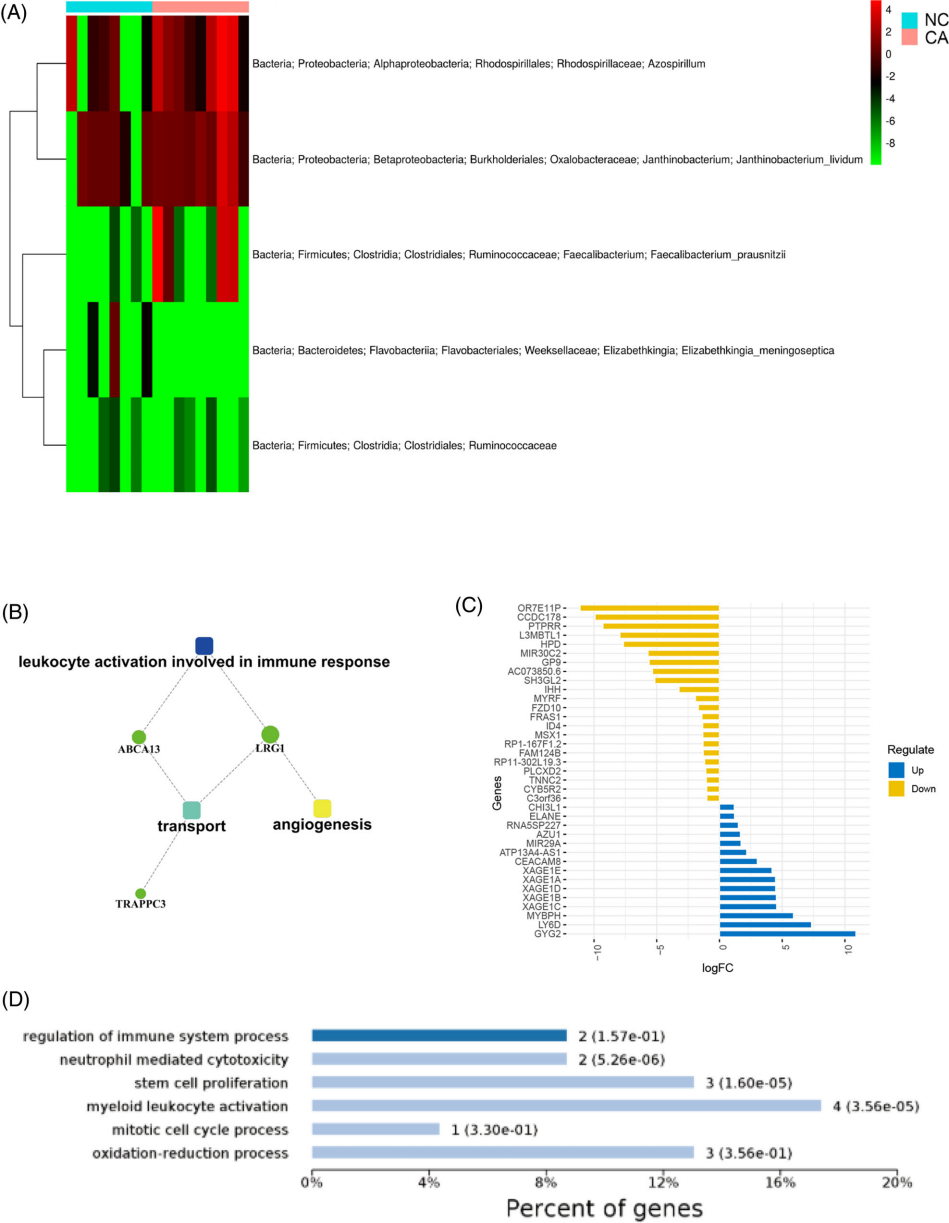
The specific microbiota and species in pGGO tissues: (A) The heat map reflects the differential species between the pGGO sample and the pGGO paracancerous sample. (B) The GO pathways enrichment analysis of the five specific OTUs‐related DEPs. (C) The deviation plot of the 37 specific OTUs‐related DEGs, (up‐regulated DEGs: 15; down‐regulated DEGs: 22). (D) The function analysis of specific OTUs‐related DEGs by GO pathways enrichment analysis. DEGs, differentially expressed genes; DEPs, differentially expressed proteins; OTUs, operational taxonomic units; pGGO, pure ground‐glass opacity

**FIGURE 2:**
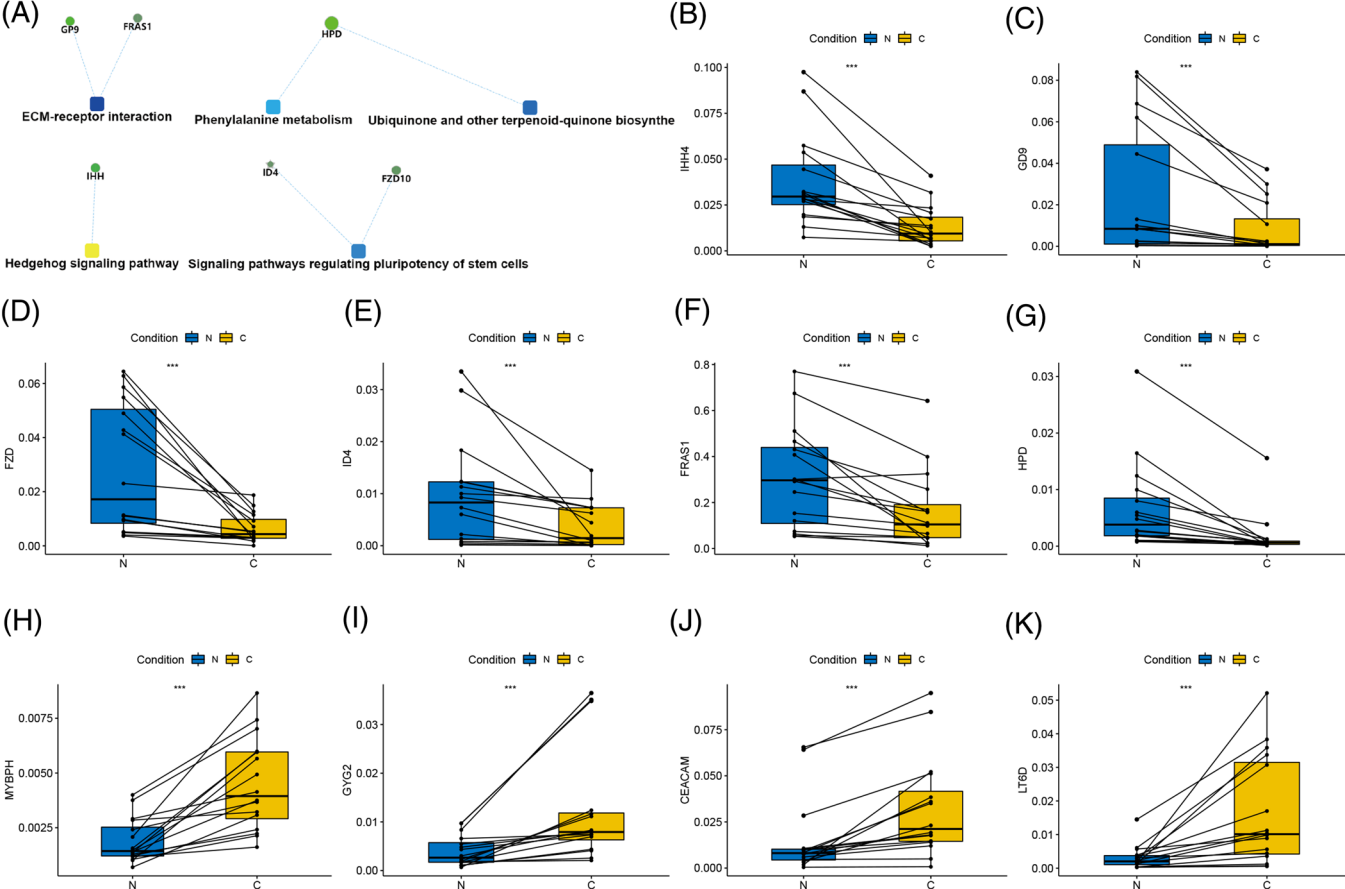
The validation of the specific OTUs‐related DEGs in pGGO tissues: (A) The construction of the function network of the specific OTUs‐related DEGs by KEGG analysis. (B–K) The validation of the specific OTUs‐related significant DEGs by real‐time quantitative PCR (up‐regulated DEGs: 4; down‐regulated DEGs: 6, ***p < .001). DEGs, differentially expressed genes; OTUs, operational taxonomic units; pGGO, pure ground‐glass opacity

The linear discriminant analysis (LDA) analysis suggested that Oxalobacteraceae and Ruminococcaceae OTUs were the specific OTUs in pGGO tissues (Figure 3A). However, there was no Oxalobacteraceae‐related literature reported in pGGO, even in lung cancer. In this research, we found three OTUs containing Oxalobacteraceae, and the GO and Kyoto Encyclopedia of Genes and Genomes (KEGG) analyses revealed that the Oxalobacteraceae‐related DEGs were mainly involved in the regulation of leukocyte differentiation term and positive regulation of immune effector process term. Among them, *IHH*, *CD36*, *GAB2* and *TGFBR2* were the core genes in immunity biological processes (Figure 3B–D; Tables S5). As previously mentioned, the role of *IHH* in tumourigenesis and development has already been discussed. For CD36, some studies had shown that *CD36* could mediate cytokine production and immunological tolerance by recognizing, engulfing and phagocytizing pathogens or pathogen‐related molecules from bacteria.^4^, ^5^ Combined with our study, *CD36* may recognize pathogens or pathogen‐related molecules from Oxalobacteraceae and trigger the initiation proliferation, invasion and metastasis of pGGO. *GAB2* also plays an important role in activating the innate immune system.^6^ *TGFBR2* is a multidirectional regulator of a variety of biological processes, and the dysregulation of *TGFBR2* in CD4^+^ T cells inhibits tissue healing and vascular remodelling, thus preventing cancer development and leading to the death of cancer cells from hypoxia.^7^ With the deepening of research on tumour immune mechanisms, ‘tumour‐promoting inflammation’ had been reported as a cancer enabling characteristic. And from our study, it suggested that Oxalobacteraceae may display the ability to modulate the tumour microenvironment by influencing the core immune/inflammation‐related genes, thus having an impact on pGGO development. The Oxalobacteraceae‐related DEPs were significantly enriched in positive regulation of canonical Wnt signalling pathway, response to cytokine stimulus and metabolic‐relative pathways (Figure 4A–C; Table S6). Thus, the Oxalobacteraceae‐related DEPs were mainly involved in cell division‐, migration‐, inflammation‐ and immune‐related processes. Canonical Wnt signalling pathway had been identified for its role in carcinogenesis, and *PSMD2* had been demonstrated that involved in the Wnt signalling pathway; besides, *PSMD2* has also been demonstrated that associated with the metastatic phenotype and poor prognosis in lung cancers.^8^

**FIGURE 3:**
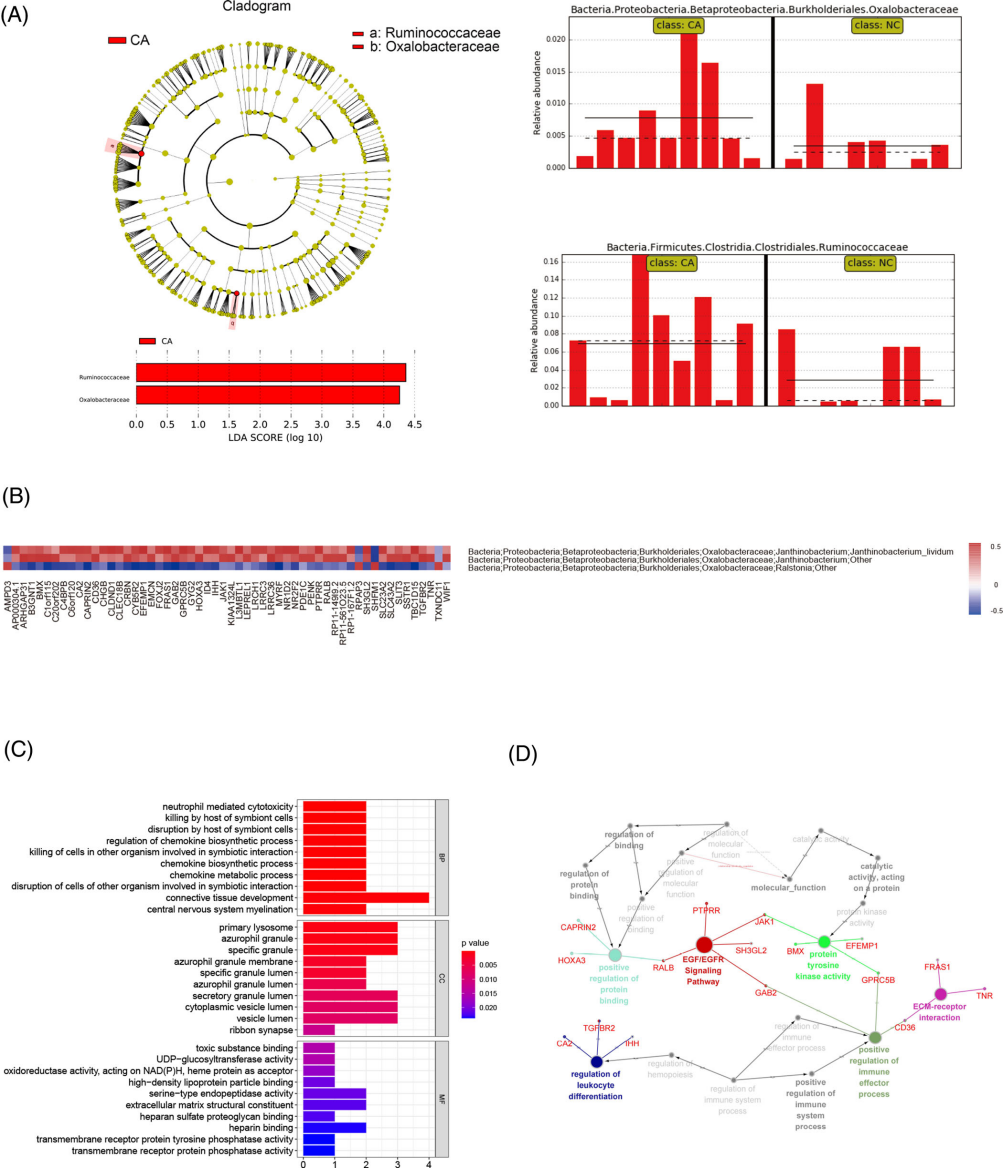
The function analysis of the Oxalobacteraceae‐related DEGs. (A) The LDA scores were obtained by linear regression analysis (LDA). The larger the LDA scores, the greater the influence of species abundance on the different effects. (B) The heat map reflected the expression of the Oxalobacteraceae‐related DEGs in three Oxalobacteraceae‐related OTUs. (C) The GO pathways enrichment analysis of Oxalobacteraceae‐related DEGs. (D) The function analysis network of Oxalobacteraceae‐related DEGs is based on the KEGG pathway enrichment analysis (different colours represent different pathways). DEGs, differentially expressed genes; OTUs, operational taxonomic units; pGGO, pure ground‐glass opacity

**FIGURE 4:**
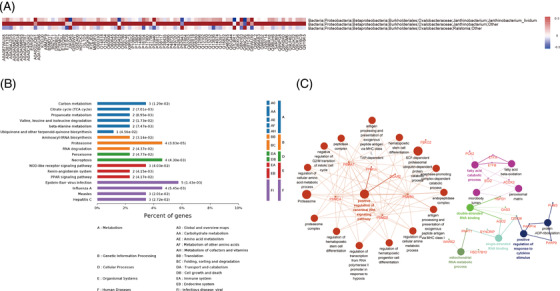
The function analysis of the Oxalobacteraceae‐related DEPs. (A) The heat map reflected the expression of the Oxalobacteraceae‐related DEPs in three Oxalobacteraceae‐related OTUs. (B) The GO pathways enrichment analysis of Oxalobacteraceae‐related DEPs. (C) The function analysis network of the KEGG pathways enrichment according to Oxalobacteraceae‐related DEPs (different colours represent different pathways). DEPs, differentially expressed proteins; OTUs, operational taxonomic units; pGGO, pure ground‐glass opacity

In conclusion, the microbiome in pGGO was mainly involved in immunosuppression, inflammation and angiogenesis processes through the Hedgehog signalling pathway. In particular, the Oxalobacteraceae was the specific microbe in the pGGO samples. And Oxalobacteraceae may display the ability to modulate the tumour microenvironment by influencing the core immune/inflammation‐related genes. This study novelly exhibited that pGGO microbiota may serve as a microbial marker and contribute to immunosuppression, inflammation development and tumour differentiation in the pGGO.

## CONFLICT OF INTEREST

The authors declare that there is no conflict of interest.

## Supporting information

Figure S1

Supporting Information

Table S1

Table S2

Table S3

Table S4

Table S5

Table S6
