# Supplementary figures and images for: Metagenomic analysis of the microbiome of lung adenocarcinoma with pure ground‐glass opacity
Source: Clin Transl Med. 2022 Jan 21;12(1):e698. doi: 10.1002/ctm2.698 (PMC8778636; doi:10.1002/ctm2.698)

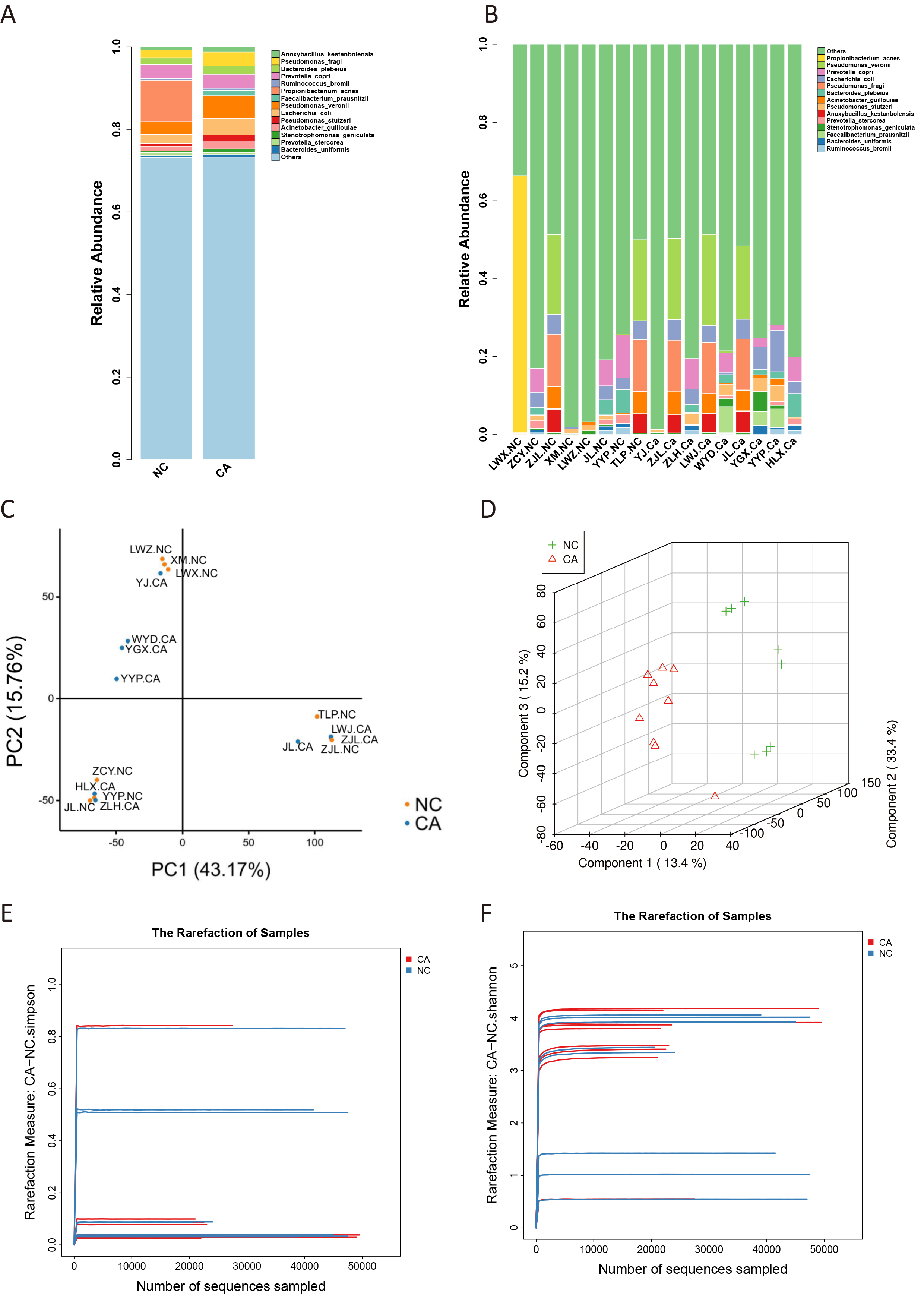

Supplement: Supplementary file 1 — Figure S1 [file CTM2-12-e698-s004.tif]
