# Supplementary material for: Metagenomic analysis of the microbiome of lung adenocarcinoma with pure ground‐glass opacity
Source: Clin Transl Med. 2022 Jan 21;12(1):e698. doi: 10.1002/ctm2.698 (PMC8778636; doi:10.1002/ctm2.698)
Supplement: Supplementary file 2 — Supporting Information [file CTM2-12-e698-s003.docx]

Figure S1: The microbiota diversity in each sample. (A-B), The relative abundance of the microbiota in each sample at the species level. (C-D), Principal component analysis from OTU classification and taxon data. The first, second, and third principal components associated with sample status (PC1, PC2, and PC3 explained 13.3%, 33.4%, and 15.2% of variances, respectively). Each dot represents a sample, the same color of the points from the same group (pGGO sample (red) and pGGO paracancerous (green); the closer the distance between the two points, the smaller the difference in community composition between them. (E-F), The Simpson curves and Shannon-Wiener curves. The highest point of the curve reflects the Shannon or Simpson index of the sample.

Table S1. The clinical data of the patients for the transcriptome analysis, proteome analysis, and metagenomic analysis.

Table S2. The bacteria OTUs data of the pGGO and pGGO paracancerous tissues.

Table S3. The 5 bacteria OTUs between the pGGO and pGGO paracancerous tissues.

Table S4. The 37 differential expressed genes were strongly associated with differential OTUs.

Table S5. Three bacteria OTUs contain the Oxalobacteraceae.

Table S6. The Oxalobacteraceae-related differential expressed genes in pGGO.

Table S7. The Oxalobacteraceae-related differential expressed proteins in pGGO.
